# Reclaiming ritual in palliative care: A hermeneutic narrative review

**DOI:** 10.1017/S1478951524001767

**Published:** 2025-01-27

**Authors:** Chrystabel Butler, Natasha Michael, David Kissane

**Affiliations:** 1 University of Notre Dame, Darlinghurst, Australia; 2 Faculty of Medicine, Nursing and Health Sciences, Monash University, Melbourne, Australia

**Keywords:** Spiritual care, palliative care, ritual, end of life, hospice, spirituality, ritualization

## Abstract

**Objectives:**

To explore the potential of incorporating personally meaningful rituals as a spiritual resource for Western secular palliative care settings. Spiritual care is recognized as critical to palliative care; however, comprehensive interventions are lacking. In postmodern societies, the decline of organized religion has left many people identifying as “no religion” or “spiritual but not religious.” To assess if ritual could provide appropriate and ethical spiritual care for this growing demographic requires comprehensive understanding of the spiritual state and needs of the secular individual in postmodern society, as well as a theoretical understanding of the elements and mechanisms of ritual. The aim of this paper is to provide a comprehensive and theoretically informed exploration of these elements through a critical engagement with heterogeneous literatures.

**Methods:**

A hermeneutic narrative review, inspired by complexity theory, underpinned by a view of understanding of spiritual needs as a complex mind–body phenomenon embedded in sociohistorical context.

**Results:**

This narrative review highlights a fundamental spiritual need in postmodern post-Christian secularism as need for embodied spiritual experience. The historical attrition of ritual in Western culture parallels loss of embodied spiritual experience. Ritual as a mind–body practice can provide an embodied spiritual resource. The origin of ritual is identified as evolutionary adaptive ritualized behaviors universally observed in animals and humans which develop emotional regulation and conceptual cognition. Innate human behaviors of creativity, play, and communication develop ritual. Mechanisms of ritual allow for connection to others as well as to the sacred and transcendent.

**Significance of results:**

Natural and innate behaviors of humans can be used to create rituals for personally meaningful spiritual resources. Understanding the physical properties and mechanisms of ritual making allows anyone to build their own spiritual resources without need of relying on experts or institutionalized programs. This can provide a self-empowering, client-centered intervention for spiritual care.

## Introduction

In 2004, the WHO included spiritual care in its guidelines for ethical palliative care (World Health Organization (WHO) 2019). While the critical importance of spiritual needs at end of life is well acknowledged (Giezendanner et al. 2017), in the milieu of postmodern multicultural societies, difficulties in defining spiritual care, as well as what it means to deliver this concretely, has hampered progress (Ferrell et al. 2016). Secularization and departure from organized religion has paralleled the rising popularity of spirituality, a construct which remains poorly understood even by those who embrace it (Schreiber 2012).

While academic research has developed several interventions to provide spiritual care at end of life, evidence of substantial benefit remains lacking (Gijsberts et al. 2019). There are, however, increasing reports of rituals emerging organically in palliative care settings, an intuitive response to spiritual needs (Weegen et al. 2020). The ritual of the “sacred pause” to acknowledge the death of a patient has spread internationally as a grassroots movement (Cunningham et al. 2019). Rituals created by individuals and teams working in palliative care reveal common themes, e.g., lighting a candle, opening a window, prayers, meditation, diaries, and meeting circles (Bloomer et al. 2023; Benbenishty et al. 2020; McAdam and Erikson 2020; Montross-Thomas et al. 2016; Nielsen et al. 2023; Running et al. 2008). Hospice patients also create personal ritualized practices, such as journaling, making/preparing death clothing, crafting gifts for loved ones, and life celebrations (Bourgeois and Johnson 2004; Butters 2021).

Ritual, a universally observed human phenomenon like dance, music, symbolism, and language, is thought to have arisen in the course of human evolution (Renfrew et al. 2017; Stephenson 2015). Rituals are deliberate, detailed, and repeated patterns of activity that reaffirm social ties, structure transitions in the life cycle, and generate meaning for human challenges and joys (Bell 1997). It is believed that organized religions emerged and evolved from ritualized practices (Lang 2024; Rossano and Vandewalle 2016). Reclaiming ritual as a fundamental human spiritual resource (Douglas 2002; Geertz 1973) may provide a critical key to addressing spiritual needs in secular contexts providing palliative care (Quartier 2010).

The purpose of this review is twofold: first, to better understand the nature of spiritual needs in postmodern, secularized society; and second, to clarify the core elements of ritual to better understand if creative use of ritual in the palliative care setting can meet those needs.

## Methodology

This narrative review uses a hermeneutic approach, seeing a liteature review as fundamentally an understanding process (Boell and Cecez-Kecmanovic 2014). A hermeneutic framework provides a theoretical foundation for a literature review as an “organic system that is constantly growing and changing” (Levy and Ellis 2006, 208), necessarily allowing for “diversions into unplanned areas” (MacLure 2005). Interactive and iterative processes are employed, based on the premise that parts are only understood from understanding the whole – and the whole is only understood from understanding parts (Schmidt 2014), underpinned by complexity theory, the study of nonlinear systems with large numbers of interacting variables (Tomas 2023). The literature review thus develops iteratively through numerous hermeneutic circles, building on each other in a recursive manner (Boell and Cecez-Kecmanovic 2014).

**Figure 1.**
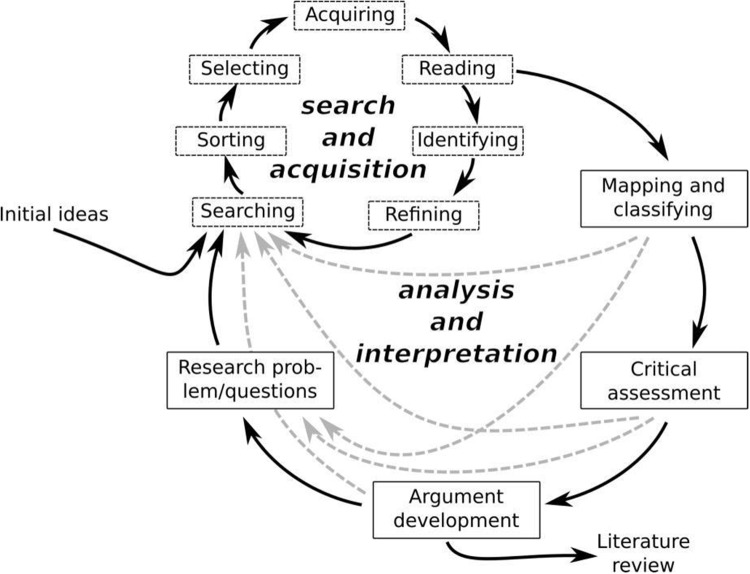
Hermeneutic framework exploring ritual.

This review employed 2 hermeneutic circles: (1) the search and acquisition circle and (2) the analysis and interpretation circle (Boell and Cecez-Kecmanovic 2014, 264), shown in Figure 1. The first circle followed the steps of searching, sorting, selecting, acquiring, and reading; the second followed the steps of analytic reading, critical assessment, mapping, and classifying. A literature review was conducted using PubMed, Web of Science, ProQuest, Sage Journals, Wiley Online Library, and Google Scholar for material written in or translated into English, with no restrictions placed on country or publication date. Search terms included the following: Ritual AND (theory, anthropology, psychology, sociology, religion, spirituality). Relevant articles were also found by scanning the references of found articles (backward search) and locating newer articles that included the original cited paper (forward search). Given the breadth of this exploratory review, specific inclusion and exclusion criteria were not used. The search was then expanded using citation pearl growing strategies following the diversions described above to terms related to ethology, evolution and performance theory, neuroscience, cognition, and child development. This process was repeated several times, until no new significant pathways were found.

The theoretical framework in which our review is situated as one that recognizes psychosocial complexities (Greenhalgh and Papoutsi 2018) along with the limitations of researching something as complex as spirituality and ritual. A synthesis of the nature, structure, value, cultural evolution, and significance of ritual developed through the following conceptual framework: (1) Religion and spirituality in Western postmodern culture; (2) Rise of secularism and decline of ritual in Western Christendom; (3) Cultural self and mind–body experience; (4) Western academic study of ritual; (5) Defining Ritual; and (6) Current understandings of ritual.

## Results

### Religion and spirituality in Western postmodern societies

The newest major religious group reported in national censuses of high-income Western cultures is “no religion” (Inglehart RF 2020). Membership in organized religions in postmodern Western societies has been in decline for decades, paralleled by the rising popularity of the construct of spirituality.

Traditionally, religion is understood as possessing substantive and functional aspects. Substantive aspects are generally conceptual in nature, systems of belief in a divine power prescribing what is important and sacred (Bruce 2011). Functional aspects are concrete and experiential, actions and behavior in pursuit of what is important and sacred, guiding humans through challenges, such as meaning-making, death, suffering, isolation, and injustice (Pargament 2001). Historically, many Christian lineages evolved emphasizing substantive mentalistic aspects and devaluing experiential practices (Elias 1998; Meyer 2012; Taylor 2007). Prevalent postmodern attitudes embrace spirituality as a reaction to and rejection of organized religion, polarizing the 2 constructs (Huss 2014; Oman 2013; Zinnbauer and Pargament 2005). Religion is commonly perceived as cognitive, impersonal, and institutionalized; in contrast, spirituality represents emotionally invested efforts to reach sacred and existential goals, finding meaning, wholeness, inner potential, and interconnections (Pargament et al. 2013).

A growing demographic in postmodern societies describe themselves as “spiritual, but not religious” (Wixwat and Saucier 2021), an emergent sociocultural phenomenon described as post-Christian spirituality. Individuals independently evolve personal credos that give meaning to existence, according to their own frame of mind, subjective experience, interests, and aspirations (Hervieu-Léger 2006). Post-Christian spirituality is characterized by 7 logically interrelated ideas: perennialism (fundamentally all religions are identical and interchangeable); bricolage (freedom to draw on different religions in a way that makes sense personally); immanence of the sacred (sacred is present in life, nature, and cosmos as an impersonal spirit, energy, or life force); cosmos (cosmos is alive, not inanimate); holism (sacred connects everything within the cosmos); self-spirituality (sacred resides within rather than without the self); and experiential epistemology (experiences and emotions are emanations of the spiritual self that lies within, inner knowing and truth) (Houtman and Tromp 2021).

In Western academic discourse, definitions for spirituality which have received wide acceptance are representative of post-Christian spirituality. Some examples are:

“The dynamic dimension of human life that relates to the way persons (individuals and community) experience, express and/or seek meaning, purpose and transcendence, and the way they connect to the moment, to self, to others, to nature, to the significant and/or the sacred” (Nolan 2011, 88).

“The aspect of humanity that refers to the way individuals seek and express meaning and purpose and the way they experience their connectedness to the moment, to self, to others, to nature, and to the significant or sacred” (Puchalski et al. 2009).

Generally, post-Christian spirituality reflects longings for embodied and transformational experiences of “connection,” the “sacred” and “transcendence” (Huss 2014). Sacrality comprises qualities of transcendence, ultimacy, and boundlessness. Transcendence is “felt” as a state outside ordinary experience; self-transcendence takes one beyond the limited confines of oneself (Otto 1926; Pargament 2011). Ultimacy is a sense of deeper truths to existence; boundlessness is an experience of feeling outside temporal and spatial constraints, merging oneself into a larger whole in which all things are interconnected (Hood 1975).

However, post-Christian spirituality remains fraught with contradictory forces. The widely cited definitions for spirituality conflate substantive, functional elements with desired consequences, which traditionally were understood as hard-won fruits of functional religious practices toward substantive goals. All-inclusive immanence erases the critical distinction between the sacred and profane (Durkheim 1912). By rejecting coherent, substantive belief systems, postmodern notions of spirituality often lack a sacred core, rendering them indistinguishable from secular pursuits, confounding yearnings for transformational experiences (Zinnbauer and Pargament 2005). Although it rejects mentalistic, dwelling Christian lineages in favor of active subjective experience, post-Christian spirituality often cannot adequately inform functional pursuits toward God; aspirants mystically *think* of themselves as *passive* vessels of the divine that need only to open to the sacred, again a mentalistic approach (Weber 1922/1963, p. 326). Finally, individualization of sprituality can exacerbate disconnectedness (Taylor 2007).

Traditionally, communal rituals provided the embodied, experiential functional aspect for religions. However, in postmodern Western societies, the historic trajectory of secularization spurred a rejection and decline of rituals.

### Rise of secularism and decline of ritual in Western Christendom

Secularization of Western societies evolved over centuries following The Reformation and Philosophies of Enlightenment; conceptions of human existence transitioned from religious worldviews of a mysterious and transcendent divine cosmos to theories of “natural law” based exclusively on the immanent, material plane. Philosophies of Enlightenment promulgated individual autonomy, rationality, and social reforms. Interlocking changes in economic, religious, technological, and political realities led to decline and derision of ritual, deemed an impediment to human progress (Taylor 2007).

The Reformation granted superiority to direct relationship and responsibility to God, as an individualistic moral self; spiritual practices transitioned to “inner dwelling” contemplative modes of self-examination and self-discipline. Collective social rituals were discouraged as disorderly and depraved; religious aesthetics and sensorial experiences were demoted and condemned (Stephenson 2015; Taylor 2007).

In The Age of Reason, from late 17th century, scientific enquiry rose to prominence and was heralded as key to human flourishing. Humans could now control and manipulate nature for advancement. The primacy of divine assistance receded, replaced by prominence of science. Descartes’ theory split reality into spirit and matter thereby creating a mind–body dichotomy. Nonmaterial, cognitive consciousness was elevated, while somatic and emotional experience denigrated. By subtracting embodied experience, humans could operate as rational, goal-oriented agents (Elias 1998; Taylor 2007).

The Romantic movement arose in rebellion against oppressive self-control and rationality. The unfettered nature of man is celebrated as a self-generative force of life/God, connection to the divine accomplished through focusing on inner experience, imagination, and self-expression, an inner quest to find a true self. The individualistic self becomes “non-porous,” a “buffered identity” (Taylor 2007). Secularization transferred religious life out of bodily forms of ritual, worship, and practice; religion becomes more and more residing in the head.

Decline in communal rituals contributes to endemic disconnectedness in human society, an erosion of embodied interrelatedness (Durkheim 1912; Sennett 2012, 2017). Modernity, by turning away from ritual, has turned away from “innate, embodied intelligence and know-how” for coping with the challenges of being human (Stephenson 2015, 5). A text from Confucian literature from early China, *Liji*, is a social cosmology describing the fall of humans from a state of harmony and well-being. The story tells that profound people appeared bringing devices, practices, and guides for recovering well-being. *Li*, as practices of ritual and ceremonies, are envisioned as knots, binding society together, connecting people to natural goodness and harmony (Stephenson 2015).

### Culture, self, and mind–body experience

Taylor (2007) proposed the concept of “social imaginary,” the way that people envision society and organize internal and external relationships. Cultural neuroscience provides evidence that self-experience arises through socially imagined templates for reality. The human neonate is born profoundly undeveloped; sense of a coherent self is cocreated through interaction with caretakers. The infant learns shared patterns for perceptual awareness as caretaker directs attention to notice certain stimuli and sensation.

Merleau-Ponty (1964, 13) illustrated the primacy of perception as the ground of experience: “The *perceived world* is always the presupposed foundation of all rationality, all value and all existence.” Perception is *prior* to and the prerequisite to experience itself, involving an active meaning-making process of selecting, organizing, and interpreting sensations (von Glasersfeld 1995). The interplay between social transmission and the functional organization of the nervous system has been described as “enculturation of brain and embrainment of culture” (Han 2013; Han and Northoff 2009; Northoff 2021). The socially imagined becomes the experience of reality (Allen and Friston 2018; Miller and Clark 2018; Roepstorff et al. 2010).

Cultural templates as an interpretive frame for understanding the world can be oriented toward independent or interdependent modes of self-experience (Gardner et al. 1999), resulting in differences in the development of neural circuits, notably for patterns of perception, sense of self, connectedness, and empathy (Chiao et al. 2009; Chiao and Mathur 2016; Marsh 2018).

Centuries of socially imagined cultural patterns in the West have emphasized and encouraged self-contained individualism as well as a decoupling of conceptual thought from embodied states, leading to an inversion of perception and experience (Malafouris 2019; Meyer 2012): “The logic of inversion, characteristic of modernity, manifest as an attempt to reconfigure the relational matrix of the world we live into a series of internal representational schemata of which our actions are but an outward expression: Through inversion, beings originally open to the world are closed in upon themselves, sealed by an outer boundary or shell that protects their inner constitution from the traffic of interactions with their surroundings” (Ingold 2010, 355).

The cultural entrainment of mentalistic religions and cognitive inversion lead to endemic disembodied disconnectedness. Cultural neuroscience shows that mind–body patterns/habits create the mind–body experience. Rituals, as mind–body practices, have evolved with human cultures as transformational technologies, manifesting embodied knowledge and wisdom.

In modern medicine, the effectiveness of placebo and nocebo treatments show that “ritual and words” can powerfully alter neurophysiology through behavioral conditioning, directing attention, expectations, and hope (Benedetti et al. 2011; Petrovic et al. 2005; Wager et al. 2004). Cultivation of a porous interdependent self has been linked to resilience, sense of safety, and reduced neural reaction to physical pain in those who feel they have social support (Coan et al. 2006; Eisenberger et al. 2007). The Tibetan Buddhist training in “vast mind” appears to prevent post-traumatic disorders (Lewis 2013, 2020). Embodied practices and rituals appear to alter perceptual reality through altering neural pathways (Winkelman 2021), suggesting that self-transcendence is a universal human capacity (Hood and Chen 2005; Piedmont 1999).

### Historical Western understandings of ritual

Historically, the Western academic study of ritual emerged from observing foreign cultures, where activities were dismissed as illogical and primitive (Bell 1997; Stephenson 2015). Durkheim (1912) pointed out the existence of secular rituals in large, complex Western societies. Rituals were social actions that expressed and reestablished the collective consciousness, as a fundamental organizing mechanism. Ritual manifests cultural symbols to provide a shared focal point for collective cohesion and meaningful existence. Rituals shape how we see, feel, and think about the world through a shared system of intersubjective symbols (Geertz 1973, 1974). Rituals encode meaning for cultural participants through experiential enactment of symbolic representations (Geertz 1973, 1974; Turner 1979, 1990).

Academics began to study ritual as performances, observable in small social conventions to grander ceremonies marking special occasions (Turner 1979, 1990). Ritual as pragmatic action establishes centrality of body and materiality. The body-as-performative shifts between representational and symbolic, a sociocultural object mediating immateriality and materiality (Csordas 2002; Grimes 2004; Schechner 2017). Communal rituals act to dissolve psychic separation between people and states of consciousness; transformation becomes possible in this “sacred liminality” (Turner 1978). Transitions in human life and death are supported through enacting 3 stages of ritual: separation from everyday life, liminal-in-betweenness and reincorporation back into society as a different self (Tambiah 1990; Van Gennep 2019).

Goffman (2017) examined how performances of everyday “interaction rituals” organize social behavior. Collins (2014) identified the necessary components for ritual interaction as: 2 or more people in physical presence; a mutual awareness and a shared focus of attention; an activity or particular symbol; and, a common emotional mood. These combined elements lead to rhythmic coordinated behavior as the foundation for group bonding, cooperation, and shared meaning-making.

Ritual as “performance,” requires adherence to scripted behavior; autonomous choice is surrendered in favor of collective action. Rappaport (1999, 24) defined ritual as “the performance of more or less invariant sequences of formal acts and utterances not entirely encoded by the performers.” One is not fully the author or owner of one’s actions, as behavior and experience are imbued by intersubjective meanings external to the performer (Laidlaw and Humphrey 2006). Thus, ritual action melts individual barriers, creating porosity and unification.

Ritual, like theater, is set apart from normal reality by “framing” the event, delineating an “as-if” space where ordinary time and context are suspended, creating liminal spaces. Performers and audience take their roles, and a shared subjunctive space is created. Combining aesthetic forces and mimetic play, actors, and audience are impacted and transformed alike as they enter the imaginal frame of “as if” together (Schechner 2017).

### Defining ritual

While ritual defies neat definitions, fundamentally it creates relationship, bringing together parts into wholes, unifying elements into embodied and experiential dynamics (Houseman 2006; Tambiah 1990). Rituals act to establish, affirm, mirror, resolve, transform, and alchemize multidimensional relationships between self, other, and cosmos. Once created and sustained, rituals become entities unto themselves, “worlding the world,” enacting “intra- and interwoven beingness,” creating social and personal realities (Mika et al. 2020, 20).

The “family resemblance” model can be used to understand ritual (Snoek 2006). By collating common elements, a general framework emerges (Table 1).

**Table 1.** Component dimensions of ritual

Temporal	Context	Content	Outcomes
Marks special events	Cultural customs and traditions	Symbolism understood	Communicates liminal or transcendent experience
Points of transition in the life cycle	Family, social, or community groups	Scripted behaviors, speeches, dance or music, multimedia	Meaningful celebration
Reused across generations	Religious community	Formalized by dress, lighting, incense	Unites community that shares
Anticipated and desired as important	Political events	Creates reverence and channels emotion	Celebrates a rite of passage

### Current understandings of ritual

Universality of ritual extends beyond humans as ethologists have long documented ritualized behaviors throughout the animal world (Huxley 1966; Stephenson 2015). In animals, ritualization occurs through exaptation, when behaviors take on functions other than the original evolutionary adaptation, evolving into communicative functions (Petak 2022). Ritualization communicates emotional states; behaviors become exaggerated, rhythmic, stylized, and stereotypical (Rossano 2009). Ritualized behavior in primates promotes socialization by overriding strong fight or flight survival reactivity; the neurophysiological state is calmed, availing opportunities to build trust, reinforce social relations and group harmony (Lang and Kundt 2023; Rossano 2009).

These neurophysiological effects of ritualization are believed to have driven biological and cultural evolution of humans (Rossano 2020; Stephenson 2015). Research shows socialization of the human child is achieved through ritualized interactions between mother and infant (Nagy and Molnar 2004). Caretakers mirror the embodied experience of the infant, using patterned, simplified, stereotypical, repetitive, exaggerated, and elaborated signals to establish communication through multimodal voice, gesture, and somatic interactions (Dissanayake 2004, 2022). Directing “joint attention” to an external object or phenomena simultaneously builds cognitive and linguistic communication skills through relational bonding (Greenspan and Shanker 2007; Tomasello et al. 2005). The ritualized patterns established between caretaker and infant become templates for interaction and bonding in wider society (Collins 2022).

Play, another universally observed behavior, underpins ritualization activity. Both humans and animals recognize signals framing transitions between “real” mode to “as-if” play mode. Play becomes prominent in caretaker–child interactions when predictable patterns become boring for the developing infant (Dissanayake 2017). Novel/creative permutations of ritualized features are used to manipulate expectation, producing a range of emotionally induced learning experiences.

Play creates capacity for “as-if” representation, driving development of conceptual and symbolic cognition, upon which ritual practice and religious thought are predicated (Burghardt 2018; Dissanayake 2017; Huizinga 2003; Renfrew et al. 2017). It is hypothesized that play activities which produced impactful, transformative experiences were repeated and formalized through ritualization (Jones 2020; Morgan 2018). Rituals and rites emerged by embedding these behaviors within ceremonial and symbolic elements, heightening their emotional impact and memorability (Bell 1997). Rites producing powerful, transformative experiences led to beliefs and values which became formalized into organized religions (Burghardt 2018). Thus, the interrelatedness of creativity, play, and ritualization coalesce as a ground from which meaning-making and world views emerge from embodied experience (Morley 2017).

Creativity underpins activities of play and ritualization and is believed to be an evolutionary capacity to flexibly adapt to changing contexts, involving tolerating ambiguity, uncertainty, and an openness to experience (Bateson and Martin 2013). Metaphorical and symbolic thought enhance creativity, merging conceptual, imaginary, intuitive, and somatosensory realms (Brown 2008). Many scholars have proposed both creativity and mystical transcendent experience involve dissolution of the bounded self, a “melting of the physical boundaries of flesh and the psychic boundaries of feeling” as transformational forces move between consciousness and the unconscious (Brown 2010, 335).

Creative endeavors have been found to enhance existential meaning and purpose, as well as transformation and healing (Nelson and Rawlings 2007; Thomson and Jaque 2019). “Distributed creativity” describes creative endeavors in groups, where dynamic interaction of ideas takes place relationally. Engaging in coordinated creative activities has been found to release oxytocin, enhancing bonding; coordinated action and gestures can increase social cooperation, empathy and positive evaluation of others (Thomson and Jaque 2019).

Neurobiologically, creativity is enhanced through interconnectedness of brain regions, maximally realized through recruiting sensory and motor systems together (Dietrich and Zakka 2023). Neuroaesthetics, the study of emotional response to beauty, such as awe or wonder, exhibits overlapping features with transcendent and transliminal experiences (Thomson and Jaque 2019). Access to spiritual transcendent states appears to require embodied engagement in execution of creative tasks (Mistrík 2011). Models for the creative process closely resemble ritualization phenomena, looping through discursive and reiterative frameworks, e.g., repeating, redefining, redirecting, reconstructing, and reinitiating. Thus, participating in embodied ritual practices can support transformational experience (Thomson and Jaque 2019).

Post-Christian spirituality reflects yearnings for transformational experience; however, lack of a coherent cosmology or substantive sacred core confounds this longing (Partridge 2021). Identifying what is sacred in life provides the focal point around which structures for meaning and purpose coalesce (Pargament et al. 2017). Through intentional action, the sacred is realized through the “world of ordinary objects experienced in a particular way” (Jones 2014, 61), a “window is opened through which people can envision a deeper reality” (Eliade 1959, 12). Sacralization, as the identification of what is sacred, transmutes the material to immaterial, connecting heaven to earth (Pargament and Mahoney 2005; Pargament et al. 2017). Once an object or activity is transformed in the mind of the perceiver, it takes on spiritual vitality, the capacity to soothe, inspire, comfort, empower, connect people to past and future, generate meaning, and engender feelings of wisdom and wholeness (LaMothe 1998; Pargament et al. 2016; Walsh 2015).

Sanctification, creation of the sacred, refers to things that are set apart, made special, through psychological, social, institutional, cultural, and situational forces (Pargament et al. 2017). Theistic sanctification involves perceiving aspects of material reality as manifestations of God; nontheistic sanctification involves identifying which aspects of life are important and therefore special (Taves 2013). Ritualization represents a process through which ordinary actions become non-ordinary, a distinctive quality of action that can be brought forth through intentional actions (Bell 1997; Laidlaw and Humphrey 2006).

Dissanayake (2009, 2018) proposed that humans manifest an innate process for transforming the ordinary through “artification,” a ritualization process arising from creativity and play developed in human caretaker–infant interactions. The ordinary and mundane is made “special,” as various aspects are patterned, simplified, made stereotypical, exaggerated, and elaborated. Focusing attention and intention toward making something “special” imbues the particular material/immaterial experience or object with value, the process through which ritual nurtures and births the sacred (Dissanayake 2013).

### Discussion: Mechanism of ritual

Rituals represent mind–body technologies for transformational experiences. Innate human capacities of play, creativity, adaptation, and communication form the basis for ritual building. Ritual is a “structuration of sensory perception and cognition in which particular human potentialities both of experience and of meaningful construction may be formed … in which the dynamics of cosmological, social and personal construction – dynamics as a field of force – achieve their most intense concentration” (Kapferer 2004, 37). Powerful effects of ritual, involve multidimensional levels of subjective and intersubjective being, intertwining cognitive, emotional, and physiological experience (Malafouris 2019), are described variously as “collective effervescence” (Durkheim 1912), “sensational forms” (Meyer 2012), and “dynamic force” (Langer 1953).

Kapferer (2004, 2006) suggested that rituals provide a technology of virtuality, establishing alternative spaces and states of consciousness, which allow us to step out of the “actuality” of lived lives into the “really real.” Ritual mechanisms slow down actuality, creating simplified, rarefied spaces, where we can enter into contained, imaginal “as-if” frameworks, enabling participants to break free from everyday life in order to reorient to everyday life. In ritual virtuality, we enter into self-reflection and intentional forces of production, construction, and reinvention, reordering and recreating our world realities (Kapferer 2004, 2006).

Through framing, ritual is set apart from the mundane (Bell 1992; Durkheim 1912; Humphrey and Laidlaw 1994). Framing can be achieved through behavior contradicting normal intention–causality linked actions (e.g., lighting candles when it is not dark); framing actions signal entry into the world of symbolic representation (Clay and Tennie 2018; Whitehouse 2021). Intentionally directing awareness to symbolic representations manifest a “ritual stance,” where “if people define something as real then it is real in its consequences” (Kapferer 2004, 40). Embodied practices invest ritual space with value, creating a tangible construction in which one can dwell (Bell 1997; Franchetto 2020; Smith 1998; Tuan 1977). Embodied meanings emerge through repeated body movements which bring awareness to certain aspects of human experience (Bourdieu 1985). Ritual is the practice of making the invisible visible, by offering multiple media for materializing the sacred. Once made material, “the invisible can be negotiated and bargained with, touched and kissed, made to bear human anger and disappointment” (Orsi 2012, 147).

Thus, ritual activity is a symbolic process, expressing complex abstractions and concrete realities with economy and simplicity, creating “blended” spaces and states, transporting and transforming consciousness (Franchetto 2020; Langer 1953). Contradictory phenomena can be brought together, reordered, readjusted, and resolved through manipulating symbolic representations (Handelman 2004). Collective rhythmic movement, dance, music, and voice create neurophysiological entrainment, linking limbic systems, blurring physical boundaries, and manifesting increased empathy and bonding (Merker et al. 2009; Perry et al. 2021). Even the simple birthday celebration blends the embodied with the intentional, emotional, and relational: gustatory experience, collective rhythmic coordinated singing, a specific and special number of candles lit and blown out with a heartfelt wish/intention.

In ritual, use of multimodal sensorial devices can act powerfully through aesthetics, an embodied experience unifying feeling and form (Langer 1953). Aesthetic forms achieve their potency through organizing dynamics of the perceptual field. “Sensational forms” offer strong stimuli, mobilizing, and training certain senses to invoke emotions, pulling together various sense impressions into a “whole” (Meyer 2012). Sensation authenticates spiritual concepts; the ritualized body becomes the producer, transmitter, and receiver of an *actual* experience of the sacred and transcendent (Meyer 2012).

Ritual could be considered a staged drama of culturally assembled embodied processes, creating bonded liminal states through joint attention, shared actions, emotional investment, and collective intentionality (Malafouris 2018). Framing of “performance” empowers observation of what is meaningful (Scheff et al. 1977). Playing a role or watching a role being played engenders self-reflexivity, driving metacognitive reflection, the ground from which understandings and meaning for one’s life are discovered (Scheff et al. 1977). Emotional and behavioral regulation is facilitated vicariously, through contained enactment of powerful emotional experiences. In performance, speech acts accomplish social magic, (e.g., wedding vows), providing a powerful vehicle for transforming “actuality” through virtuality. Practice and performance of intentional actions can cultivate different levels of consciousness which can grant access to numinous and metaphysical experience (Raposa 2004; Schilbrack 2004). Ritual stance is to invite “intentional non-intentionality,” relinquishing individualism, suspending rational and logical control, embodying a state of heightened openness; one can become porous, merging with multifaceted experience and other beings (Laidlaw and Humphrey 2006; Pehal and Cieslarová 2011). Embodied action of ritual in subjunctive spaces offers mysterious mediations, allowing connections, transformations, and permutations of relationships.

### Post-Christian spirituality and ritual in palliative care

As nondenominational rituals increasingly emerge in palliative care settings, a small number of empirical studies have studied their impact. The studied ritual activities reflect post-Christian spirituality, exhibiting individual self-spirituality, perennialism, bricolage, immanence of the sacred, and experiential epistemology.

Most of the studies document use of ritual by staff and medical professionals. Jones (2005) found hospice workers used personal rituals for self-care. Personal rituals are used to better manage grief by marking transitions, connections, affirming the value of the individual (Balmer et al. 2022; Doka 2002). Small, ritualized activities created structure, meaning, and value for patients and carers; were a source of human connectedness, compassion; and achieved a common value of a “good death” (Weegen et al. 2020). Montross-Thomas et al. (2016) reported over 70% of hospice staff and volunteers used personally meaningful rituals after the death of their patients to help them cope. Those who used rituals demonstrated significantly higher compassion, satisfaction, and significantly lower burnout. Kapoor et al. (2018) found 79% of respondents felt ritual brought closure and helped them overcome the feelings of disappointment, grief, distress, and failure after the death of their patient. For 73%, ritual instilled and encouraged a sense of team effort; and for 82% ritual made efforts feel appreciated.

Running et al. (2008) reported using organized grief rituals in a work setting reduced stress, job dissatisfaction, and compassion fatigue among hospice staff. Established rituals, such as the sacred pause or lit candle after a death, supported reflection and processing of repeated deaths (Mayr et al. 2024; Volek 2021).

There is much less research on rituals used by patients. For hospice patients and carers, personally derived rituals organically emerge (Quartier 2010); and ritualization of daily routines intensify as death approaches (Weegen et al. 2020). Personally meaningful practices provide a mechanism where all involved can feel less isolated, more supported, discovering opportunities for intimacy, reconciliation, and closure (Bourgeois and Johnson 2004; Bowman et al. 2000). The symbolism of ritualized meaningful practices affords those involved to seek understanding and meaning, promoting spiritual comfort (Loseth 2002; Prior 1999). Personally constructed rituals have been found to positively support bereavement and heal complicated grief of survivors (Lewis and Hoy 2011; Reeves 2011; Sas and Coman 2016).

### Implications for secular palliative care settings

In postmodern multicultural societies, institutionalized palliative care presents diverse demographics. The power of ritual in undercutting differences and conflict is achieved through its universality and ambiguity (Seligman and Weller 2012). The unspoken embodied action of ritual allows individuals to derive their own meaning and value.

For those who are dying, personalized rituals provide more support. Identifying what is important and valuable in that individual’s life establishes a sacred core which informs the construction of ritual building. The dying individual and significant people can join into a creative process employing symbology, sensorial embodied action, aesthetics to honor, and celebrate the life lived.
